# Indoor Dining and In-Person Learning: A Comparison of 30 US Cities

**DOI:** 10.3390/ijerph182010967

**Published:** 2021-10-19

**Authors:** Gabriella O’Leary, Alina S. Schnake-Mahl, Vaishnavi Vaidya, Usama Bilal, Jennifer Kolker

**Affiliations:** 1 Department of Health Management and Policy, Drexel University Dornsife School of Public Health, Philadelphia, PA 19104, USA; geo34@drexel.edu (G.O.); jak682@drexel.edu (J.K.); 2 Urban Health Collaborative, Drexel University Dornsife School of Public Health, Philadelphia, PA 19104, USA; vgv24@drexel.edu (V.V.); ub45@drexel.edu (U.B.); 3 Department of Epidemiology and Biostatistics, Drexel University Dornsife School of Public Health, Philadelphia, PA 19104, USA

**Keywords:** COVID-19, indoor-dining, schools, policy, US

## Abstract

With limited US federal leadership on closing and re-opening strategies to mitigate the COVID-19 pandemic, cities and states were left to enact their own policies. This article examines two key sets of policies—in-person learning in public elementary schools and indoor dining—across 30 of the largest US cities in the summer, fall, and winter of 2020. We review indoor dining and in-person elementary education policy decisions between 1 May 2020 and 14 December 2020 across 30 US cities. We review the public health evidence, political power, and jurisdictional challenges that cities faced, and the policy implications of these factors. Overwhelmingly, indoor dining re-opened in cities while in-person elementary schools were kept closed; indoor dining re-opened in all cities in fall 2020, while only 40% of public elementary schools re-opened for in-person instruction. Looking ahead to fully bringing students back for in-person learning, and considering future potential community outbreaks, this retrospective analysis can help inform city and state governments on policy decisions around indoor dining and reopening/closing schools for in-person learning.

## 1. Introduction

Throughout 2020, the United States (US) was not consistent in their guidance or implementation of COVID-19 policies across the country. From stay-at home orders to testing protocols, and absent federal policies [1], cities and states implemented various strategies at different times to prevent the spread of COVID-19. In-person learning in elementary schools and the closure and re-opening of indoor dining are two key policies that reflect important social and economic values, and that cities and states were left to determine policies around. This article compares the re-opening of in-person learning in public elementary schools and indoor dining re-openings throughout 2020 across 30 of the largest and most politically left-leaning cities in the country. The cities in this study are members of the Big Cities Health Coalition (BCHC), an organization comprised of the largest metropolitan health departments in the US.

In-person learning is key for children’s educational, social, and developmental benefits [2]. Early childhood education is a strong predictor of subsequent educational, socioeconomic, and health outcomes and schools are often the primary place where lower-socioeconomic students receive food and other essential services [3,4]. Additionally, academic success is a primary indicator for overall well-being and adult health outcomes [4,5]. Children living in poverty, children of color, English-language learners, and those with disabilities are disproportionately impacted by school closures [2]. Early research found that students suffered academically and emotionally from remote learning; elementary school students experienced significant learning loss in math, and these losses were even greater for children of color [5]. Students of color are more likely to live in remote-only school districts but are less likely to have the resources to navigate online education including access to computers and internet [6].

In September 2020, when school districts were making re-opening decisions, evidence from other countries was just beginning to emerge on potential COVID-19 outcomes in schools and amongst young children [3,7,8] and many school districts, teachers, and families feared in-school transmission if schools re-opened for in-person instruction [9]. Since existing levels of community spread impact the degree to which COVID-19 spreads in schools [3,7], low transmission in European and Asian schools may have reflected the low community transmission rates in those countries [3], rather than demonstrated general in-school transmission risk. In fact, COVID-19 transmission in school districts in the US that implemented comprehensive mitigation measures reflected that of the general community [10]. However, appropriate personal protective equipment (PPE) and funding for testing and contact tracing were not available in many US schools throughout the fall, elevating risk of COVID-19 exposure for many students and teachers. Resource availability to safely re-open schools followed existing patterns of racial segregation and school divestment; schools in low-income areas were more likely to lack adequate funding to follow strict COVID-19 protocols, and often needed updates to ventilation systems to mitigate risk of COVID-19 spread [11].

In September 2020, there was also little research available on the impacts of indoor dining on COVID-19 rates, though the mechanisms of dining indoors (indoor, difficult to wear masks while eating, multiple households) suggested it was likely a high-risk activity [12,13]. The collapse of the restaurant industry also had potentially damaging consequences for the economy, including declining jobs and tax revenue for cities. Additionally, at the time, Congress had not passed an additional stimulus package, leaving restaurants without an economic safety-net.

Limited data in summer/fall 2020 made school re-opening decisions difficult. A year later, there is now some evidence of COVID-19 spread in schools, but transmission appears to be limited, particularly for pre-school and elementary aged children, especially when mitigation efforts such as mask mandates, student pods, and social distancing measures are put in place, and when community rates are low [3,8,10,14]. Notably, some studies highlight increased community transmission following school reopening and some find no increases [7,14,15,16,17] but the evidence suggests that COVID-19 spread can be managed through implementation of school-based mitigation strategies [11]. For example, a study on incidence and secondary transmission of SARS-CoV-2 infections in schools found that in the first 9 weeks of in-person instruction in North Carolina, there were low rates of secondary transmission in schools, which the authors attributed to mitigation strategies such as mask wearing and physical distancing [18].

In comparison, evidence now clearly suggests indoor dining is a high-risk activity and opening dining is associated with increased spread of COVID-19 [13,19]. Research has found substantial decreases in COVID-19 case rates in cities that kept dining closed compared to those that re-opened, regardless of capacity restrictions [13]. Additionally, a CDC study found that states allowing on-premise dining had an increase in daily COVID-19 case growth rates 41–100 days after implementation of the policy and an increase in daily growth rates 61 to 100 days after, indicating that restricting on-premise dining can help limit community transmission of COVID-19 [20]. In a review of 20 studies examining the role of hospitality venues in driving COVID-19 rates, researchers found that indoor dining was a major site of super spreader events and posed a high risk for COVID-19 spread; closing indoor dining is one of the most effective ways to reduce the spread of COVID-19 [13,21].

The purpose of this article is to compare two important policy issues in parallel by systematically reviewing indoor-dining and in-person elementary school policy decisions in some of the largest and most progressive-leaning cities in the country in the fall and winter of 2020. We hypothesized that large cities would re-open schools for in-person learning before re-opening indoor dining. To analyze this hypothesis, we present descriptive data from the 30 largest cities in the US. To our knowledge, this analysis of re-opening decisions in cities across the country is unique, and highlights under-recognized policy factors including pre-emption, jurisdictional power challenges, and political pressures that impact health policies and have consequences for public health. As a new school year begins and we consider endemic COVID-19 and future pandemics, a retrospective analysis of policy decisions can inform future responses.

## 2. Materials and Methods

We compare the re-opening of in-person learning in public elementary schools and indoor dining re-openings between 1 May 2020 and 14 December 2020 across the 30 study cities. These dates were selected to reflect when restaurants in many cities first re-opened indoor dining, following early spring closures, and when schools went on winter break in late fall 2020. We focus on cities to highlight local heterogeneity in re-opening decisions, as there are substantial differences in the scope of local governments’ authority to regulate indoor dining and re-opening of public schools. While some cities and states re-opened middle and high schools for in-person instruction in addition to elementary schools, we focus on public elementary school re-openings because the long-term social and health impacts of in-person elementary learning are clearly stated in the literature and elementary school children have had the hardest time with remote learning [3,5]. We define re-opening for in-person learning as bringing back the majority of public elementary school students for in-person instruction, including schools that brought students back part-time (i.e., hybrid learning models). Several public-school districts such as Baltimore, Boston, and San Diego only brought back for in-person instruction a small number of students with the greatest needs, including English-language learners, students who were homeless, and students with disabilities. Because these school districts did not bring back broader groups of students, we did not categorize these schools as re-opened for in-person learning, until they brough back the majority of students. We define re-opening indoor dining as any dining inside of a restaurant, with or without capacity restrictions. We collected information on statewide and city/county orders by searching multiple publicly available databases and state/city websites listing these orders, reviewed state/city orders, and identified public statements by searching news articles, Twitter posts, and state/city websites (see Appendix A).

## 3. Results

### 3.1. Spring and Summer

**Figure 1:**
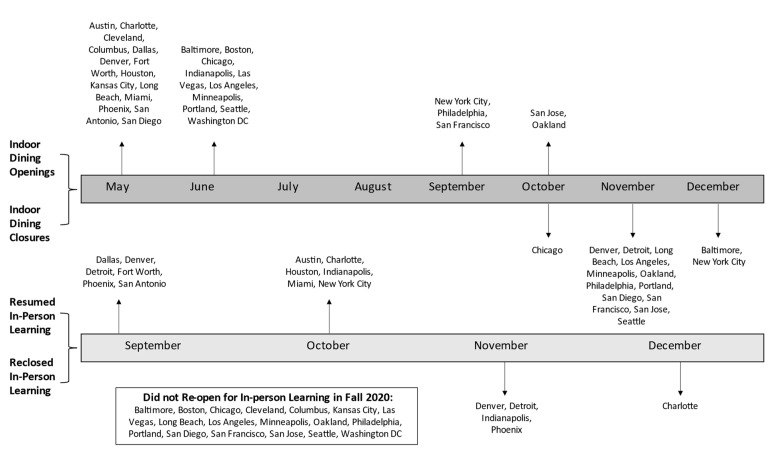
Indoor dining and in-person learning opening and closing timeline.

Schools in all 30 cities closed for in-person instruction after the initial outbreak of COVID-19 in March 2020. In-person elementary schools did not re-open in any of the 30 cities until September 2020, when in-school learning re-opened in 6 (20%) cities. At this time, indoor dining had already re-opened in 25 cities. By the end of October, indoor dining had re-opened in all 30 study cities, while public elementary schools were open for in-person learning in only 12 (40%) cities. Figure 1 illustrates the varying re-opening and re-closing indoor dining and in-person learning schedules in study cities throughout 2020.

### 3.2. Early Fall

**Figure 2:**
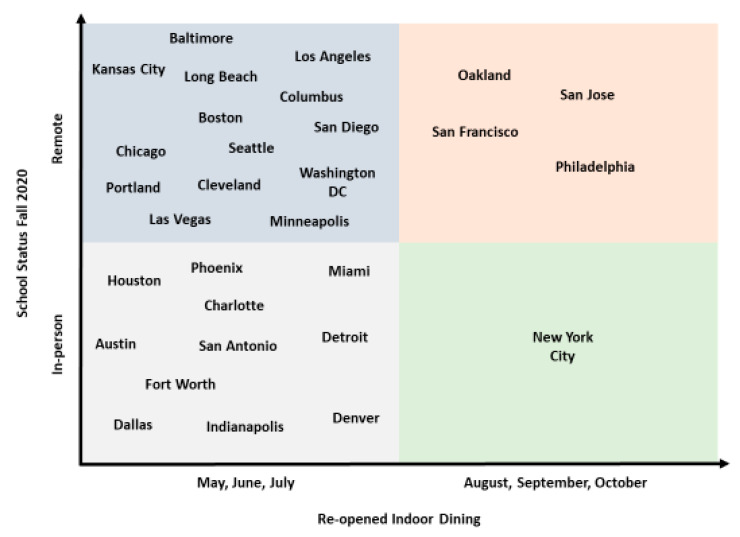
Status of indoor dining and in-person learning in 30 large US cities in the early fall of 2020 (by 14 October 2020).

Figure 2 shows that by the end of October 2020, indoor dining had reopened in all 30 study cities. In most of these 26 (86%) cities, indoor dining re-opened in late spring/summer, while in only 5 (16%) cities—Philadelphia, New York City, San Francisco, Oakland, and San Jose—dining was not re-opened until the fall. In comparison, in-person public elementary schools re-opened in only 12 (40%) cities. Some public-school districts such as Charlotte and Indianapolis initially resumed in-person learning in early fall and later switched to virtual learning due to increasing case rates (see Appendix A).

### 3.3. Late Fall/Early Winter

**Figure 3:**
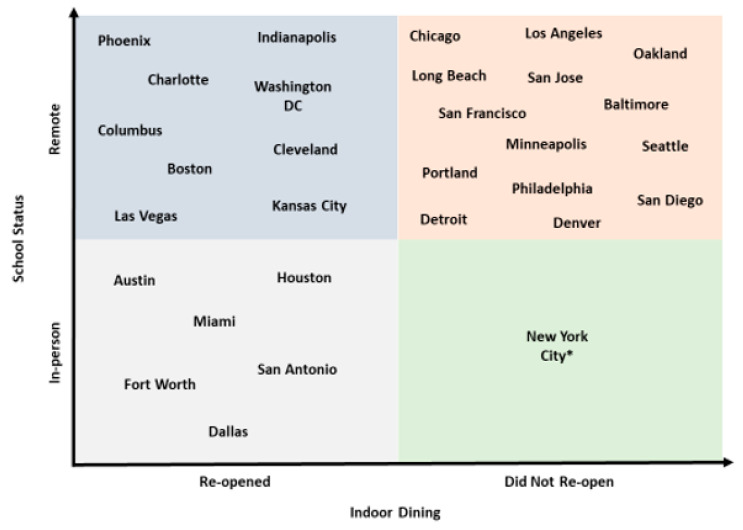
Status of indoor dining and in-person learning in 30 large US cities in the late fall of 2020 (by 14 December 2020). * New York City public schools closed for in-person instruction on 19 November 2020 but reopened for elementary school students on 7 December 2020.

By Thanksgiving, indoor dining closed again in many cities and schools switched back to virtual learning due to increases in COVID-19 cases. By late December, in-person learning closed in an additional 5 (16%) cities and indoor dining closed in 15 (50%) cities. As a result, in 14/20 (46%) cities, both indoor dining and in-person classes were closed by the end of December; dining was kept open but schools were closed in 10 (33%) cities; both indoor dining and in-person learning were permitted in 6 (26%) cities; and in just 1 city, schools were kept open for in-person learning while indoor dining was closed (See Figure 3). New York City was the only city where indoor dining was closed but schools stayed open; public schools closed for in-person instruction on 19 November 2020 but re-opened for elementary school students on 7 December 2020 [22].

## 4. Discussion

Indoor dining re-opened in fall 2020 in all 30 large US cities in our study, but in-person learning resumed in public elementary schools in only 12 (40%) cities. By late December, indoor dining was permitted in 15 (50%) cities and in-person elementary learning was allowed in only 7 (23%) cities. Contrary to our hypothesis, re-opening dining was more common in cities across the country than re-opening in-person learning. While previous research and reporting showed that schools can re-open safely for in-person learning when mitigation strategies are put in place and that indoor dining contributes to the spread of COVID-19, this study fills a gap in national analysis of school and indoor dining policy decisions in cities across the US. Additionally, we discuss the jurisdictional powers, political pressures, and policy implications of these decisions, which can inform future policy decisions.

### 4.1. Jurisdictional Power: Public Schools and Dining Closure/Re-Opening Decisions

There is substantial variation in jurisdictional power to close and re-open public schools. Due to absent federal policies, decisions varied by city and state, and included: state-ordered closures by executive order, state-ordered regional closures, school district closures, hybrid/remote instruction only, and state-ordered in-person instruction by executive order [23]. The majority of states left re-opening decisions to local discretion and individual schools or districts [23], but states including Texas and Florida ordered in-person instruction, while California ordered regional school closures [24]. Even locally, decision making authority on schools varies. In cities such as New York City, the mayor unilaterally makes decisions, while in other cities, such as Philadelphia, there is an independent school board.

Authority to determine indoor dining re-opening and re-closing also varied across the cities. Often, the states, not cities, made indoor dining re-opening or closing decisions as part of state-led phased re-opening or re-closing plans. In some states (e.g., Texas and Arizona), state governments employed government pre-emption ceilings, a legislative doctrine whereby a higher level of government prohibits a lower level, such as city governments, from enacting a program or policy that puts in place stricter restriction requirements than the state [25]. Pre-pandemic, states pre-empted local governments on laws including minimum wage and anti-gun legislation [25] and during the pandemic, governors employed ceiling pre-emptions in various ways, including to warn cities such as Austin to rescind local emergency orders that imposed stricter COVID-19 restrictions [26]. For example, by the end of December, of the 15 cities where indoor dining was permitted, 7 cities were pre-empted by the state from keeping indoor dining closed. For indoor dining, some states used a form of pre-emption called a regulatory floor, which allowed city discretion on taking additional actions, beyond what was required by the state, to protect residents [26]. For example, while Philadelphia qualified to re-open indoor dining on 26 June 2020 per the state’s re-opening guidelines, city officials restricted indoor dining until 8 September 2020. Finally, some states did not explicitly pre-empt cities around indoor dining, allowing city discretion to determine indoor dining re-openings.

### 4.2. Political Pressure

Teachers’ unions also played a crucial role in negotiating school closures and re-openings [9]. Fearing unsafe working conditions, many teachers resisted resuming in-person learning, pressured governors to shut down schools across states, and pushed for hazard pay and improved safety measures [9,27]. Additionally, families played an important role in decisions to resume in-person learning; many parents, particularly low-income and parents of color, opted to keep their children home [28]. These decisions reflect inequities in school funding, recognition that schools serving predominantly low-income or children of color often lack the resources necessary to make returning to school safe, and that COVID-19 has disproportionately impacted communities of color [6,11,28]. Historical distrust of the public school system’s ability to maintain safe buildings and communicate transparency was also important as families weighed the risks and benefits of sending their children back for in-person learning [28].

The restaurant industry pushed to keep indoor dining open, through lobbying efforts and lawsuits against mayors and governors [29]. For example, the Oregon Restaurant and Lodging Association sued Governor Kate Brown over her two-week ban on indoor dining, but Federal judges rejected the case, ruling that closing indoor dining was done for a plausible reason and within the state’s authority [30]. Of note, and as opposed to the situation with schools, to our knowledge, there are few unions that have fought for the safety of restaurant workers who may be exposed from indoor dining.

### 4.3. Policy Implications

Decisions to re-open dining and schools throughout 2020 were partially based on what evidence was available at the time, and since then, additional research demonstrates strong evidence of COVID-19 spread when indoor dining re-opens, while re-opening elementary schools for in-person learning is likely not a major contributor to community spread [13,16]. The CDC now states that re-opening K-12 schools for in-person instruction this fall is a national priority, and schools can safely re-open by promoting vaccinations and with consistent use of prevention strategies such as masks, physical distancing, and ventilation [16]. Very few large cities in the US re-opened schools for in-person learning in fall and winter of 2020, as many policymakers overestimated the risk of transmission in schools and underestimated the risk posed by indoor dining.

President Biden has made re-opening schools for in-person instruction a national priority since taking office in January 2021 and has stated that all schools should resume in-person instruction by fall 2021 [31]. To help re-open schools, he signed several executive actions including reimbursing schools for PPE; increasing testing for students and teachers; expanding vaccine capacity to include equitable distribution to teachers; and improving data collection and contact tracing in schools [32]. The USD 1.9 trillion American Rescue Plan Act of 2021 provided USD 130 billion to cover costs related to hiring staff, upgrading ventilation systems, and increasing testing measures [32]. Teachers in all states are eligible for the vaccine, adding an additional layer of protection; however, there is still uncertainty on when vaccinations will open for elementary-aged children.

While COVID-19 rates remained high throughout spring 2021, there were declining hospitalizations and deaths across the US due to vaccination efforts [33]. Yet, while restaurants returned to full capacity and cities fully reopened in most states, many students remained in remote or hybrid instruction in spring 2021 and schools did not return to in-person learning until fall 2021 [34]. This winter, with the Delta variant and outbreaks across the US, states and cities may again need to decide how to control COVID-19, forcing decisions on policies such as in-person learning or indoor dining.

According to the CDC, for schools to stay open and open fully, community transmission rates need to stay low, below 5 per 100,000 persons within the last 7 days [16]. Restricting and closing non-essential indoor spaces such as restaurants [7], ideally with an economic safety-net in place for the industry, is one vital policy to limit community transmission levels and make schools safer for in-person instruction in communities experiencing surges in hospitalization rates. Federal, state, and local funding will continue to be necessary to allow cities and states to close indoor dining, and limit restaurants to lower-risk dining options if there are new outbreaks. Providing sufficient funding for underfunded schools, usually located in low-income neighborhoods, which have borne the worst consequences of the pandemic [35], will be critical to allowing schools to stay open safely while reducing parents’ concerns of in-school exposure.

Lastly, state pre-emption prevented indoor dining from staying closed in many cities. The COVID-19 pandemic highlights that state pre-emptive ceilings can hinder public health efforts and worsen health inequities, when cities are prohibited from enacting policies that protect public health. Moving forward, cities need the ability to enact policies that are more protective, as the US is likely to see continuing endemic COVID-19, particularly in communities with vaccination gaps.

### 4.4. Recommendations

As we shift to endemic COVID-19, this study presents important implications for policy and practice. As cities remove indoor dining restrictions, there are several strategies that can be put in place to safely re-open restaurants for indoor dining and reduce community spread. For example, restaurants can mandate vaccines, provide adequate ventilation and outdoor dining options, make modifications to promote physical distancing, and offer flexible sick leave policies to employees [36]. Similarly, as public schools across the US open for in-person instruction, mitigation strategies such as vaccine mandates, masking, ventilation, and testing protocols should be put in place to stop the spread of COVID-19 [10,16]. Federal funding is key to execute these mitigation strategies, especially as some states withhold funding from school districts that implement mask mandates [37]. Additionally, the COVID-19 pandemic has led to severe learning setbacks for students across the US, particularly for low-income children and racial/ethnic minorities. Moving forward, state and city governments should create plans to ensure academic success for all [38]. Future research should also assess indoor dining and in-person school policy decisions in spring and fall 2021 and consider the impacts of other policy decisions such as vaccine and mask mandates in schools and indoor restaurants on COVID-19 spread.

### 4.5. Limitations

We define reopening for in-person learning as bringing back the majority of public elementary school students for in-person instruction. Some school districts brought back a small number of students with the greatest needs, and these were not categorized as re-opened for in-person education in our study. We define re-opening indoor dining as any dining inside of a restaurant, with or without capacity restrictions, though capacity restrictions varied across the study cities. However, research found substantial decreases in COVID-19 case rates in cities that kept dining closed compared to those that re-opened, regardless of capacity restrictions [21], suggesting our simplified categorization represents important policy variation. The study is limited to 30 cities across the country, so implications may not be generalizable to non-metro areas. However, the cities represent over 62 million people and the study includes diverse large cities across the country.

## 5. Conclusions

While education and re-opening schools are national priorities, this was not apparent in realized policy decisions. Indoor dining re-opened in fall 2020 in all 30 large US cities in our study, but in-person learning resumed in public elementary schools in only 12 (40%) cities. By late December, indoor dining was permitted in 15 (50%) cities and in-person elementary learning was allowed in only 7 (23%) cities. These decisions were complicated by a web of jurisdictional power struggles, political pressure, and imperfect information about the risk of COVID-19 spread in schools and from indoor dining.

Looking forward to the 2021–2022 school year, endemic COVID-19, and future possible pandemics, city and state governments should carefully assess the trade-offs between activities that are critical for society (e.g., schools) versus non-essential businesses and leisure activities, such as indoor dining. In-person learning is critical for children’s educational and social development and the evidence now clearly states that closing indoor dining venues is an effective measure to reduce COVID-19 incidence and mortality rates. This retrospective analysis can help inform future policy decisions around indoor dining and school closures/reopening and help us understand past decisions.

## Data Availability

Data available in Appendix A.

## Appendix A. City Indoor Dining and Public Schools Re-Opening/Closure Dates and Sources

**City Indoor Dining and Public Schools Re-Opening/Closure Dates and Sources**				
**City**	**Indoor Dining Re-Opening/Closing Dates**	**Public School Re-Opening/Closing Dates (2020–2021 School Year)**	**Indoor Dining Sources**	**School Sources**
Austin	1/5/2020	5/10/2020	Governor Abbott Issues Executive Order Relating To The Expanded Reopening Of Services|Office of the Texas Governor|Greg Abbott (accessed on 14 December 2020)	3_V 4.2 Open for Learning_Revised 111120.pdf (austinisd.org) (accessed on 14 December 2020)
Baltimore	19/6/2020–10/12/2020	Did not resume in-person classes	Baltimore Allows Indoor Dining June 19 2020 MAYORAL EXECUTIVE ORDER (baltimorecity.gov) (accessed on 14 December 2020)	https://www.baltimorecityschools.org/sites/default/files/2020-08/DraftReopeningPlan-8.14.2020.pdf https://www.baltimorecityschools.org/small-group (accessed on 14 December 2020)
Boston	22/6/2020	Did not resume in-person classes	Reopening Massachusetts: Baker-Polito Administration Initiates Transition to Step Two of Second Phase of Four-Phase Approach|Mass.gov (accessed on 14 December 2020)	Boston Public Schools Announces Delay in Next Phase of In-Person Learning Due to Newly Released Public Health Data Boston Public Schools Shifts to All Remote Learning Due to Rising COVID-19 Cases Citywide (accessed on 14 December 2020)
Charlotte	22/5/2020	12/10/2020–14/12/2020	Governor Roy Cooper Executive Order May 20 2020 Charlotte Re-opens Indoor Dining along State Guidance (accessed on 14 December 2020)	CMS to return to in-person instruction in phased plan CMS will return to remote learning until Jan. 19 (accessed on 14 December 2020)
Chicago	26/6/2020–30/10/2020	Did not resume in-person classes	BeSafe.Capacity-Limitations-City-of-Chicago-Phase-4-Guidelines.pdf Microsoft Word-CDPH Order 2020-11 Reissued 10.30.20 FINAL (chicago.gov) (accessed on 14 December 2020)	Chicago Public Schools to Begin School Year with Full Remote Learning Model Based on Public Health Guidance and Parent Feedback|Chicago Public Schools (cps.edu) (accessed on 14 December 2020)
Cleveland	21/5/2020	Did not resume in-person classes	COVID-19 Update: Reopening of Restaurants, Bars, and Personal Care Services|Governor Mike DeWine (ohio.gov) (accessed on 14 December 2020)	10/16/2020-CEO Update on 2nd Quarter Recommendations (clevelandmetroschools.org) (accessed on 14 December 2020)
Columbus	21/5/2020	Did not resume in-person classes	COVID-19 Update: Reopening of Restaurants, Bars, and Personal Care Services|Governor Mike DeWine (ohio.gov) (accessed on 14 December 2020)	CCS Announces Second Quarter Learning Model (ccsoh.us) (accessed on 14 December 2020)
Dallas	1/5/2020	28/9/2020	Governor Abbott Issues Executive Order Relating To The Expanded Reopening Of Services|Office of the Texas Governor|Greg Abbott (accessed on 14 December 2020)	Dallas ISD Schools To Reopen A Week Early For Students Moving Between Certain Grade Levels–CBS Dallas/Fort Worth (cbslocal.com) (accessed on 14 December 2020)
Denver	27/5/2020–20/11/2020	28/9/2020–30/11/2020	Restaurant Reopening Statement (denvergov.org) Denver Moves to Level Red on State’s Revised COVID-19 Dial (denvergov.org) (accessed on 14 December 2020)	Our DPS Weekly: Planning for Safe, Gradual Return of Kindergartners, 1st-Graders|Denver Public Schools (dpsk12.org) Our DPS Weekly: Important Update on Return to School|Denver Public Schools (dpsk12.org) (accessed on 14 December 2020)
Detroit	8/6/2020–11/18/2020	8/9/2020–11/16/2020	Whitmer-Governor Whitmer Rescinds Safer at Home Order, Moves Michigan to Phase Four of the MI Safe Start Plan (accessed on 14 December 2020)	DPSCD Suspension of Face to Face Learning FAQ Nov 16 2020 v2 (detroitk12.org) (accessed on 14 December 2020)
Fort Worth	1/5/2020	5/10/2020	Governor Abbott Issues Executive Order Relating To The Expanded Reopening Of Services|Office of the Texas Governor|Greg Abbott (accessed on 14 December 2020)	Fort Worth ISD to start transition to in-person learning next week; schedule released (msn.com) (accessed on 14 December 2020)
Houston	1/5/2020	19/10/2020	Governor Abbott Issues Executive Order Relating To The Expanded Reopening Of Services|Office of the Texas Governor|Greg Abbott (accessed on 14 December 2020)	2020–2021 Houston ISD Reopening Plan/Reconnect Safely, Return Strong-Home (accessed on 14 December 2020)
Indianapolis	1/6/2020	5/10/2020–23/11/2020	455ab1988f7f491684889f6200d1ce1e.pdf (citybase-cms-prod.s3.amazonaws.com) (accessed on 14 December 2020)	IPS Students Start Returning to the Classroom in Phased-In Approach—*Indianapolis Public Schools Newsroom* (ipsnewsroom.org) IPS Will Return to 100 Percent Remote Learning, Starting Nov. 23.—*Indianapolis Public Schools Newsroom* (ipsnewsroom.org) (accessed on 14 December 2020)
Kansas City	15/5/2020	Did not resume in-person classes	KM_C368-20200512093120 (kcmo.gov) (accessed on 14 December 2020)	https://www.kcpublicschools.org/reopening-kcps Reopening Notifications-Kansas City Public Schools (kcpublicschools.org) (accessed on 14 December 2020)
Las Vegas	9/5/2020	Did not resume in-person classes	2020-05-07-COVID-19 Declaration of Emergency Directive 018-Phase One Reopening (Attachments) (nv.gov)	Microsoft Word-TRANSITION PLAN FINAL RELEASE 1 11-9-2020.docx (boarddocs.com)
Long Beach	30/5/2020–25/11/2020	Did not resume in-person classes	Restaurants, Barbers and Hair Salons to Open (longbeach.gov) City of Long Beach Issues Modified Safer at Home Health Order (accessed on 14 December 2020)	LBUSD News (09/10/20) Distance Learning to Continue through Jan. (lbschools.net) (accessed on 14 December 2020)
Los Angeles	30/5/2020–25/11/2020	Did not resume in-person classes	L.A. County again closes restaurants amid coronavirus surge—*Los Angeles Times* (latimes.com) (accessed on 14 December 2020)	Latest News/Return to School-Schools Reopening List (lausd.net) (accessed on 14 December 2020)
Miami	27/5/2020–8/7/2020Re-opened again 31/9/2020	5/10/2020	Miami-Dade restaurants reopen despite coronavirus concerns|Miami Herald Statement from Miami-Dade County Mayor Carlos A. Gimenez on additional closures related to COVID-19 surge (miamidade.gov) Miami To Restart Indoor Dining After Almost 2 Months Of Closures (forbes.com) (accessed on 14 December 2020)	Miami-Dade School Board Votes to Start in-Person Learning Oct. 5–NBC 6 South Florida (nbcmiami.com) (accessed on 14 December 2020)
Minneapolis	10/6/2020–18/11/2020	Did not resume in-person classes	COVID-19 news updates/Office of Governor Tim Walz and Lt. Governor Peggy Flanagan (mn.gov) (accessed on 14 December 2020)	5 Phases to Safe Learning-MPS_CMF (mpls.k12.mn.us) (accessed on 14 December 2020)
New York City	30/9/2020–14/12/2020	1/10/2020–19/11/20Reopened again 7/12/20	Indoor Dining Returns to New York City, but Will Customers?—*The New York Times* (nytimes.com) Just When Restaurants Thought 2020 Couldn’t Get Any Worse …—*The New York Times* (nytimes.com) (accessed on 14 December 2020)	https://www.nytimes.com/2020/11/18/nyregion/nyc-schools-covid.html https://www.nytimes.com/2020/11/29/nyregion/schools-reopening-partially.html (accessed on 14 December 2020)
Oakland	23/10/2020–18/11/2020	Did not resume in-person classes	Alameda County is reopening indoor dining. Here’s how it’s going (sfchronicle.com) (accessed on 14 December 2020)	https://t.e2ma.net/message/l78mod/1jp43bh (accessed on 14 December 2020)
Philadelphia	8/9/2020–20/11/2020	Did not resume in-person classes	Philadelphia to Reopen Indoor Dining on Sept 8 (accessed on 14 December 2020)	District schools will remain fully virtual at this time–The School District of Philadelphia (philasd.org) (accessed on 14 December 2020)
Phoenix	11/5/2020	3/9/2020	https://azgovernor.gov/sites/default/files/eo_2021_0.pdf (accessed on 14 December 2020)	COVID 19—Maricopa County Regional School District (mcrsd.org) (accessed on 14 December 2020)
Portland	19/6/2020–11/18/2020	Did not resume in-person classes	Portland restaurants and bars can reopen Friday: Here, are 5 things you need to know—oregonlive.com (accessed on 14 December 2020)	Portland Public Schools Information/Fall 2020 (pps.net) (accessed on 14 December 2020)
San Antonio	1/5/2020	8/9/2020	Governor Abbott Issues Executive Order Relating To The Expanded Reopening Of Services|Office of the Texas Governor|Greg Abbott (accessed on 14 December 2020)	Aug 27 parent letter-yellow phase-final-span-eng.pdf (saisd.net) (accessed on 14 December 2020)
San Diego	21/5/2020–14/11/2020	13/10/2020	Reopening (sandiegocounty.gov) (accessed on 14 December 2020)	Phases of Reopening-San Diego Unified School District (accessed on 14 December 2020)
San Francisco	30/9/2020–14/11/2020	Did not resume in-person classes	San Francisco to Move Forward with Reopening More Businesses and Activities on September 30 (accessed on 14 December 2020)	SFUSD Updates Timeline for In-Person Learning | SFUSD (accessed on 14 December 2020)
San Jose	13/10/2020–17/11/2020	Did not resume in-person classes	Mandatory Directive: Dining, Bars, Wineries, and Smoking Lounges (sccgov.org) (accessed on 14 December 2020)	San José Unified Prepares For In-Person Instruction in January| Press Releases|What’s Happening|San José Unified School District (sjusd.org) (accessed on 14 December 2020)
Seattle	5/6/2020–16/11/2020	Did not resume in-person classes	SafeStartPhasedReopening.pdf (wa.gov) Inslee announces statewide restrictions for four-weeks|by WA Governor’s Office|Washington State Governor’s Office |Medium (accessed on 14 December 2020)	School Board Approval-Seattle Public Schools (seattleschools.org) (accessed on 14 December 2020)
Washington DC	22/6/2020	Did not resume in-person classes	DC Ready for Phase 2 Reopening Monday–NBC4 Washington (nbcwashington.com) Phase Two|coronavirus (dc.gov) (accessed on 14 December 2020)	Statement from Mayor Bowser on the Agreement with the Washington Teachers’ Union on a Return to In-Person Learning|dcps (accessed on 14 December 2020)
